# A comparative analysis of deferoxamine treatment modalities for dermal radiation‐induced fibrosis

**DOI:** 10.1111/jcmm.16913

**Published:** 2021-10-06

**Authors:** Christopher V. Lavin, Darren B. Abbas, Evan J. Fahy, Daniel K. Lee, Michelle Griffin, Nestor M. Diaz Deleon, Shamik Mascharak, Kellen Chen, Arash Momeni, Geoffrey C. Gurtner, Michael T. Longaker, Derrick C. Wan

**Affiliations:** ^1^ Hagey Laboratory for Pediatric Regenerative Medicine Stanford University School of Medicine Stanford CA USA; ^2^ Institute for Stem Cell Biology and Regenerative Medicine Stanford University Medical Center Stanford CA USA

**Keywords:** dermal fibrosis, fibrosis treatment, radiation therapy, translational science

## Abstract

The iron chelator, deferoxamine (DFO), has been shown to potentially improve dermal radiation‐induced fibrosis (RIF) in mice through increased angiogenesis and reduced oxidative damage. This preclinical study evaluated the efficacy of two DFO administration modalities, transdermal delivery and direct injection, as well as temporal treatment strategies in relation to radiation therapy to address collateral soft tissue fibrosis. The dorsum of CD‐1 nude mice received 30 Gy radiation, and DFO (3 mg) was administered daily via patch or injection. Treatment regimens were prophylactic, during acute recovery, post‐recovery, or continuously throughout the experiment (*n* = 5 per condition). Measures included ROS‐detection, histology, biomechanics and vascularity changes. Compared with irradiated control skin, DFO treatment decreased oxidative damage, dermal thickness and collagen content, and increased skin elasticity and vascularity. Metrics of improvement in irradiated skin were most pronounced with continuous transdermal delivery of DFO. In summary, DFO administration reduces dermal fibrosis induced by radiation. Although both treatment modalities were efficacious, the transdermal delivery showed greater effect than injection for each temporal treatment strategy. Interestingly, the continuous patch group was more similar to normal skin than to irradiated control skin by most measures, highlighting a promising approach to address detrimental collateral soft tissue injury following radiation therapy.

## 1 INTRODUCTION

Whether curative or palliative, half of all patients with cancer will undergo radiation therapy during their treatment course. Collateral toxicity to normal tissue is oftentimes the dose‐limiting factor and an unfortunately pervasive consequence of cancer treatment.^1^, ^2^, ^3^, ^4^, ^5^ Great advancements have been made in radiotherapy techniques such as directing radiation to tumour sites and fine‐tuning dosing regimens; however, modern radiation treatments continue to cause substantial collateral injury.^6^, ^7^ These enhanced techniques are currently the only truly effective prophylaxis against radiation‐induced fibrosis (RIF) of the skin or subcutaneous tissue. RIF can cause a multitude of detrimental outcomes, from cosmetic changes to life‐threatening conditions. Lymphedema, joint contracture, necrosis, and nonhealing wounds are just some of the many complications.^8^ Among these radiation side effects, one of the most common is radiation dermatitis, as overlying skin is invariably irradiated along with the tumour. Healthcare providers grade the severity of radiation dermatitis by its clinical presentation and potential for harm. The Common Terminology Criteria for Adverse Events Version 5.0 (CTCAE 5.0) provides a 1–5 grading scale, and dermatitis treatment is most often based on its CTCAE grade.^9^ Damage to overlying skin is twofold. Early effects, occurring during or soon after treatment, result from immediate DNA and cellular damage by ionizing radiation. Late effects arising from dysregulated tissue repair, however, may progress to become the more injurious of the two, culminating in the pathophysiologic state of RIF. Microvascular injury, activation of inflammatory cytokine cascades, and reactive oxygen species (ROS) production accentuate and perpetuate one another long after radiation exposure has ceased.^6^, ^10^ These pathologic sequelae are commonly separated temporally and grouped into ‘acute’ or ‘chronic’ changes, before and after 90 days respectively.^2^, ^11^ Ultimately, a long‐term imbalance in favour of profibrotic and pro‐inflammatory cytokines drives chronic dermatitis and RIF progression.^8^

The iron‐chelating agent, deferoxamine (DFO), has emerged as a potential therapeutic for improving RIF of the skin in murine subjects.^12^, ^13^ DFO addresses multiple pathogenic mechanisms of the fibrotic reaction within the dermis following irradiation (IR). First, iron chelation stabilizes the transcription factor, HIF‐1α, by limiting iron‐dependent degradation, in turn, upregulating downstream pro‐angiogenic growth factor production.^14^, ^15^, ^16^, ^17^, ^18^ This promotes de novo blood vessel growth in a background of endothelial destruction, while augmenting oxygen and nutrient delivery to injured skin. Second, chelation of free iron decreases oxidative damage by limiting Fenton‐based ROS generation, which is dependent on ferric iron as a catalyst.^19^, ^20^, ^21^ As inflammatory reactions self‐perpetuate in radiation toxicity, the multi‐pronged benefits of DFO administration have been shown in preclinical studies to not only reverse RIF, but also minimize the damage that would have occurred when given prior to the onset of fibrosis.^13^ Aside from symptomatic treatment, there are very few approved therapies for RIF treatment and prevention. DFO has promise as a safe, effective treatment with the potential to fill this void, but uncertainty remains regarding the best delivery route. Recently, topical administration of DFO has been studied in wound‐healing experiments.^22^, ^23^, ^24^, ^25^ The reverse micelle transdermal drug delivery system employed in these studies allows DFO, a large, hydrophilic molecule, to better permeate through the hydrophobic stratum corneum of the epidermis before dispersing in the more aqueous environment of the dermis.^22^ In contrast, a direct injection of solubilized DFO may be more efficient in mitigating radiation‐mediated dermal fibrosis since it circumvents the hurdle of epidermal penetration. In this preclinical study, we evaluated the effectiveness of these delivery modalities with a focus on the timing of DFO administration, which may provide insight as to which downstream effects of iron chelation are important for the mitigation of fibrosis.

## 2 MATERIALS AND METHODS

### 2.1 Animals

Seventy female CD‐1 nude immunodeficient mice (Crl:CD1‐*Foxn1^nu^*, Charles River) were separated into two experimental groups. Mice used for ROS‐related assays (20 mice total) were treated daily with patch or injection DFO, prior to and during IR. They were sacrificed 24 h after the final IR session. Mice used for histology, biomechanical testing, and vascularity measures (50 mice total) were treated with DFO as noted below. All procedures were performed in concordance with animal welfare and safety regulations outlined by APLAC (Protocol #31212).

### 2.2 Irradiation regimen

A lead shield with 1.5 ×2 cm rectangular cutouts was used to protect all tissue except for each mouse's dorsal skin. The skin was irradiated with 30 Gy and fractionated into six sessions of 5 Gy every other day using a Kimtron Polaris SC‐500 x‐ray machine (Kimtron Inc.). This dosing regimen would be considered a hypofractionated approach in modern clinical practice, which is associated with more severe late skin reactions than newer, altered fractionation schedules.^8^

### 2.3 Deferoxamine administration

**FIGURE 1:**
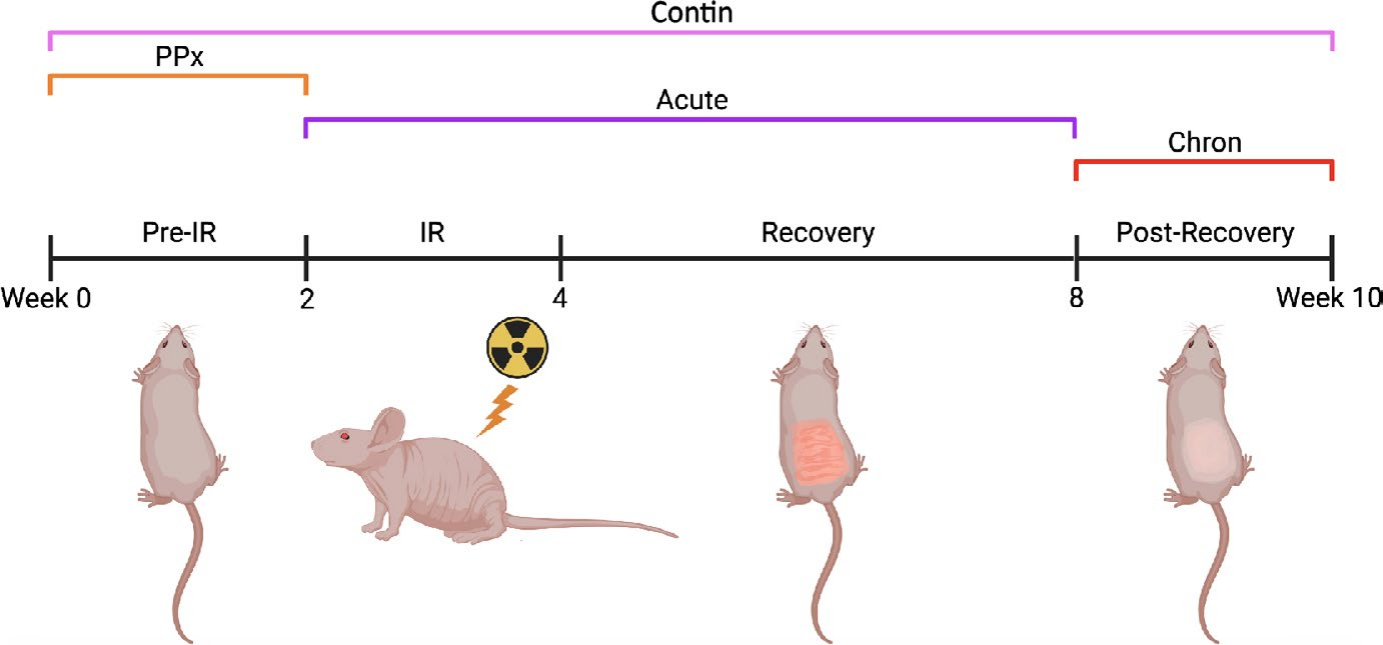
Treatment regimen schematic. Treated mice received DFO via patch or injection; both administration routes were tested within each treatment period (PPx, Acute, Chron, Contin). Abbreviations: Chron, chronic; Contin, continuous; DFO, deferoxamine; IR, irradiation; PPx, prophylactic

Deferoxamine was given as a direct injection or transdermal patch. 3 mg was given daily during each mouse's treatment period. Injections were comprised of DFO mesylate powder dissolved in sterile PBS to a concentration of 1 mg/100 μl; 300 μl was administered over the entire 1.5 × 2 cm irradiated area using a 28‐gauge syringe. DFO reverse micelle transdermal delivery patches (TauTona Group) were dosed at 1 mg/1 cm^2^; a 1.5 × 2 cm rectangle patch (3 mg total) was placed directly over the irradiated area, with a Tegaderm Film (3M) dressing surrounding the patch to fasten it in place. Mice received DFO either prophylactically prior to IR, during the acute injury phase (2 weeks IR regimen plus the following 4 weeks of recovery), during the post‐recovery chronic injury phase, or continuously throughout the entire experiment (*n* = 5 per treatment administration route and period). Treatment timelines for these mice are represented in Figure 1.

### 2.4 Tissue harvest

Irradiated skin was excised in full, immediately following euthanasia, one mouse at a time. The 1.5 × 2 cm rectangle was divided for tensile testing, with strips stored in cold PBS, histology, and with specimens preserved in 4% paraformaldehyde prior to paraffin embedding, and the remainder snap frozen in liquid nitrogen and stored at −80°C.

### 2.5 Reactive oxygen species detection

Dermal‐free iron content was quantified by staining for ferric iron (Fe^3+^) using Perls Prussian blue method (ab150674; Abcam).^26^, ^27^ Blue pixel area was obtained from 40× magnification images (*n* = 15 per condition) using the same ImageJ analysis described above but modified for blue hue detection. Oxidative stress was quantified in several ways. First, immunofluorescent staining of 8‐Isoprostane (8‐Iso) was performed. Incubation was performed with anti‐8‐Isoprostane primary antibody (1:100, MBS621657; MyBioSource) followed by an Alexa Fluor 488‐conjugated donkey anti‐goat IgG secondary antibody (1:500, ab150129; Abcam). Green pixels were quantified with a similar ImageJ analysis of 20× magnification images (*n* = 15 per condition) after manually cropping out the epidermis, which displayed high levels of 8‐Iso fluorescence in all samples. Second, an ELISA (ab138881; Abcam) was performed to quantify oxidized and reduced glutathione. Snap frozen tissue from each condition (100 mg total, pooled 20 mg per mouse) was homogenized in 2 ml of cold mammalian cell lysis buffer (ab179835; Abcam) using a Dounce homogenizer. Samples were deproteinized (ab205708; Abcam) and placed in a 96‐well, black, clear‐glass bottom microplate in triplicate to be analysed using an Infinite M Nano + plate reader (Tecan). Finally, Bcl‐2‐assosciated X protein (BAX) level was quantified by ELISA (ab233624; Abcam) and was performed in quadruplicate. Snap frozen tissue from each condition (100 mg total, pooled 20 mg per mouse) was homogenized in 2 ml of the cell extraction buffer, and the assay was performed per manufacturer's instructions.

### 2.6 Skin biomechanics

Both longitudinal in vivo and post‐harvest ex vivo mechanical testing were performed. A Cutometer Dual MPA 580 (Courage + Khazaka electronic) provided weekly measures of skin elasticity of each mouse throughout the experiment via suction and release measurement with a 2‐mm probe aperture, set to 300 m bar of negative pressure (*n* = 20 per condition: four reads per mouse weekly). After 10 weeks, final tensile testing of skin samples was performed on full‐thickness strips from each mouse. Samples were kept in PBS, on ice, until tested several hours after harvest. A Bionix 200 (MTS Systems Corporation) tensile testing machine was used, and the Young's Modulus (stress/strain) was calculated on MATLAB (MathWorks) based on the Bionix 200 software output and calliper measurements of the exact width, length and thickness of each sample. Histological preparations included haematoxylin and eosin (H + E), Masson's Trichrome (TC) and Picrosirius Red (Picro). All imaging was performed on a Leica DMI4000 B inverted microscope (Leica Microsystems). Dermal thickness, from base of epidermis to intradermal adipose, was measured directly on the imaging software (Leica Application Suite X) using 20× magnification (*n* = 25 per condition) of the H + E specimens. Collagen content was quantified from 20× magnification images (*n* = 20 per condition) of the TC slides using a binarizing RGB filter in MATLAB. Picro 40× images taken under a polarizing light source (*n* = 100 per condition) and were analysed in MATLAB with a previously described proprietary machine‐learning algorithm to determine differences in collagen ultrastructure. This algorithm compared 294 parameters including fibre dimensions, directionality, branching and maturity among many other learned variables.^28^

### 2.7 Dermal microvasculature measurements

Biweekly laser Doppler perfusion imaging on a PeriScan PIM 3 (Perimed) provided in vivo measurements of dermal blood flow to the dorsum of each mouse.^29^, ^30^ Scans were performed under inhaled anaesthesia with a heating pad below the induction chamber, while ensuring constant ambient room temperature at 73°F. Mean perfusion of the 1.5 ×2 cm treatment field was recorded twice for each mouse with back‐to‐back scans. Additionally, CD31 immunofluorescent staining was performed on histologic sections of each skin sample. Incubation was performed with anti‐CD31 primary antibody (1:100, ab28364; Abcam) followed by an Alexa Fluor 647‐conjugated donkey anti‐rabbit IgG secondary antibody (1:500, ab150075; Abcam). Red pixel area was obtained from 20× magnification images (*n* = 15 per condition) via ImageJ (NIH) analysis that recognized red hues, binarized the images and counted selected pixels.^31^

### 2.8 Statistical analysis

Analyses were performed in GraphPad Prism 9.0.0 (GraphPad Software), and statistical significance was determined based on a *p‐value <0.05. Error bars on box and whisker plots represent 95% confidence intervals. A one‐way analysis of variance (ANOVA) was performed to determine statistical significance for each measure. Tukey's multiple comparisons tests were used when examining means between two groups within an ANOVA.

## 3 RESULTS

### 3.1 Oxidative stress

**FIGURE 2:**
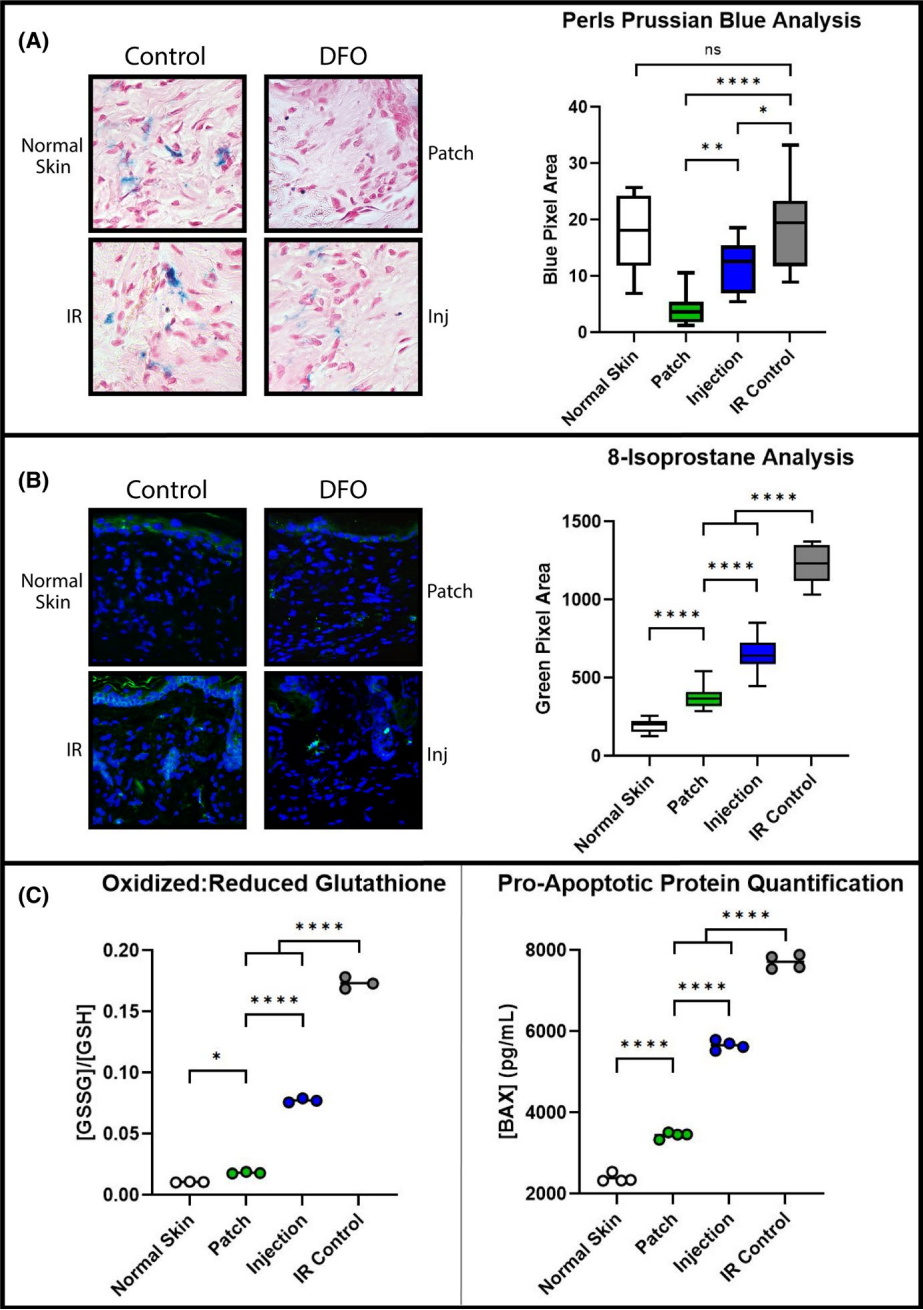
ROS and oxidative stress markers. (A) Perls Prussian Blue staining for ferric iron: representative 40× images (left) and quantification (right chart). DFO treatment decreased mean free iron in the dermis compared with IR Control and Normal Skin (*p < 0.05, **p < 0.01, ****p < 0.0001). (B) Representative 20× 8‐Iso images (left) and quantification (right chart). DFO treatments decreased ROS‐mediated lipid peroxidation compared with IR Control skin (****p < 0.0001). (C) ELISA of glutathione oxidation state (left chart) and BAX protein levels (right chart). DFO treatment decreased ratio of GSSG:GSH compared with IR Control skin (****p < 0.0001). DFO treatment also decreased BAX apoptotic protein level compared with IR Control skin (****p < 0.0001). Greater reduction in GSSG:GSH and BAX protein for DFO Patch relative to Injection were noted. Abbreviations: 8‐Iso, 8‐Isoprostane; BAX, Bcl‐2‐associated protein X; DFO, deferoxamine; ELISA, enzyme‐linked immunosorbent assay; GSSG:GSH, ratio of oxidized to reduced glutathione; Inj, injection; ns, not significant; ROS, reactive oxygen species

To compare ability for transdermal patch delivery of DFO via reverse micelles to chelate free iron relative to direct injection, mice were treated prior to and during radiation therapy with either approach. Perls Prussian Blue staining revealed DFO was effective at removing ferric iron from the dermis, with levels significantly less than irradiated skin. However, the patch chelated more iron than injection (Figure 2A). 8‐Iso levels, a marker for reactive oxygen species, rose in response to radiation injury. Immunofluorescent staining revealed DFO administration lowered quantities of 8‐iso in the dermis of treatment groups compared to IR control skin, with the patch decreasing 8‐Iso levels more than injection (Figure 2B). As a second measure of ROS, oxidized:reduced glutathione (GSSG:GSH) ratios were significantly increased following IR. Both DFO treatment groups displayed lower GSSG:GSH ratios, though again patch DFO resulted in greater active GSH relative to oxidized GSSG than injection DFO (Figure 2C). Finally, both DFO treatment groups reduced levels of p53‐regulated BAX protein compared with untreated irradiated skin (Figure 2C).

### 3.2 Dermal fibrosis

**FIGURE 3:**
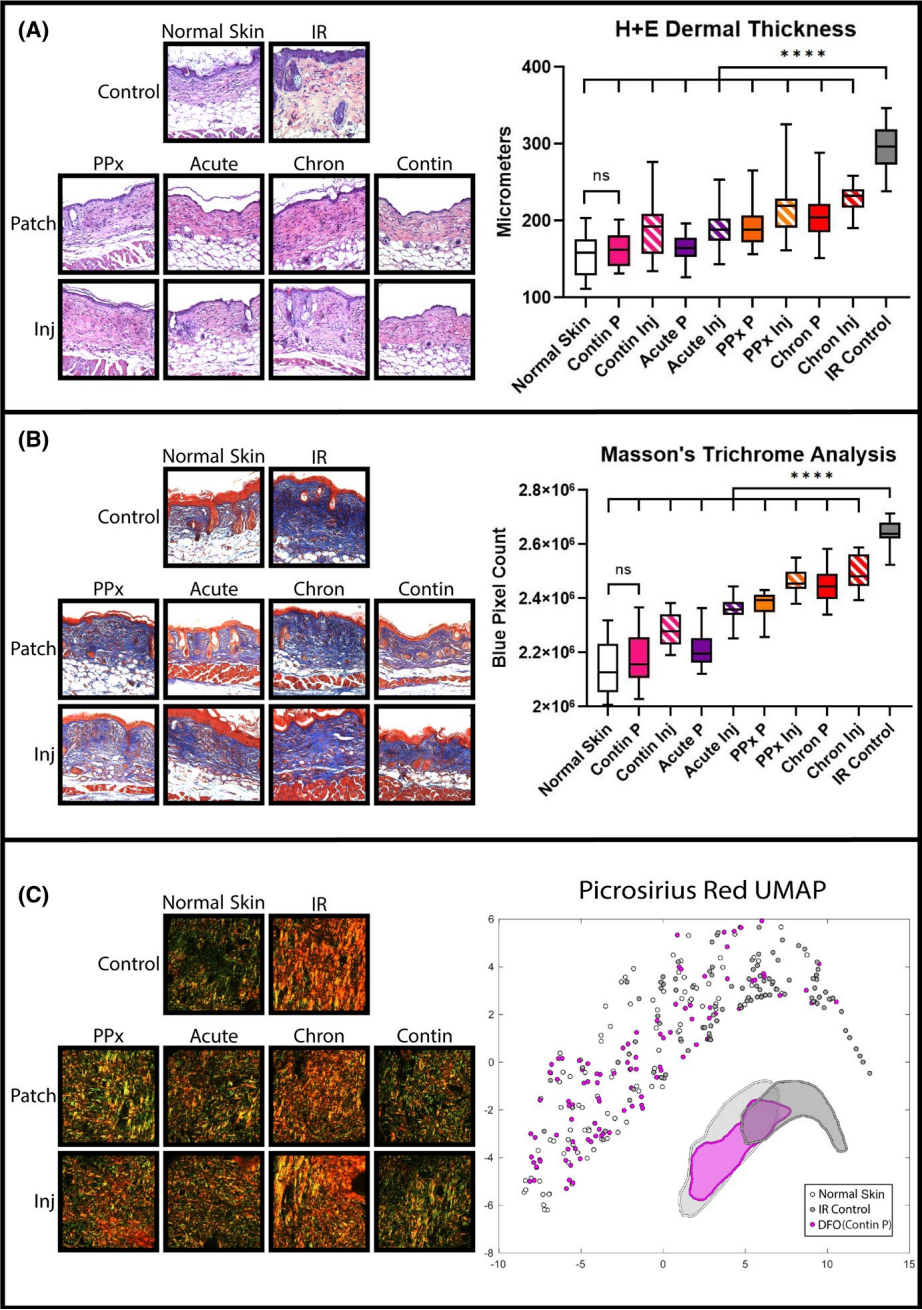
Histological RIF quantification. (A) Representative 20× H + E specimens (left) and quantification of dermal thickness (right chart). Mean dermal thickness was reduced with DFO treatment compared with IR control skin (****p < 0.0001). Greatest reduction in dermal thickness with continuous patch (Contin P) was noted, which was similar to normal skin. (B) Representative 20× TC samples (left) and quantification of collagen content (right chart). Mean collagen content was reduced with DFO treatment compared with IR Control skin (****p < 0.0001). Greatest reduction in collagen content with continuous patch (Contin P) was noted, which was similar to normal skin. (C) Representative 40× Picro images (left) and machine‐learning algorithm‐derived collagen ultrastructure UMAP representation of extracellular matrix characteristics for normal skin (light grey), IR Control (dark grey), and continuous DFO patch (pink). Normal Skin and Contin P DFO treatment overlapped more with each other than with IR Control skin, represented by cluster shape approximation underneath plot points. Abbreviations: Chron, chronic; Contin, continuous; DFO, deferoxamine; H+E, haematoxylin and eosin; Inj, injection; IR, irradiation; P, patch; Picro, Picrosirius Red; PPx, prophylactic; RIF, radiation‐induced fibrosis; TC, Masson's Trichrome; UMAP, uniform manifold approximation and projection

With the ability for both transdermal patch delivery and direct injection of DFO to reduce free iron levels and reactive oxygen species in irradiated skin, we subsequently evaluated temporally directed DFO treatment relative to radiation therapy (Figure 1). As expected, dermal thickening was appreciated on H+E specimens ten weeks after IR. DFO administration, whether through patch or injection, decreased dermal thickness compared with IR control skin when administered prophylactically, during acute recovery, in the post‐recovery chronic phase, or continuously (Figure 3A). Interestingly, continuous patch treatment (Contin P) was similar to normal skin in measured dermal thickness. Dermal collagen content, as shown by TC staining, was noted to increase following IR (Figure 3B). DFO administration decreased collagen content, and again, collagen content with Contin P was similar to normal skin (Figure 3B). Picro analysis was performed to assess RIF changes to collagen fibre assembly and ultrastructure (Figure 3C and Figure S1). We found that DFO treated groups clustered more similarly to normal skin than to IR control skin when analysed using a supervised computer learning algorithm, especially the Contin P group (Figure 3C).

**FIGURE 4:**
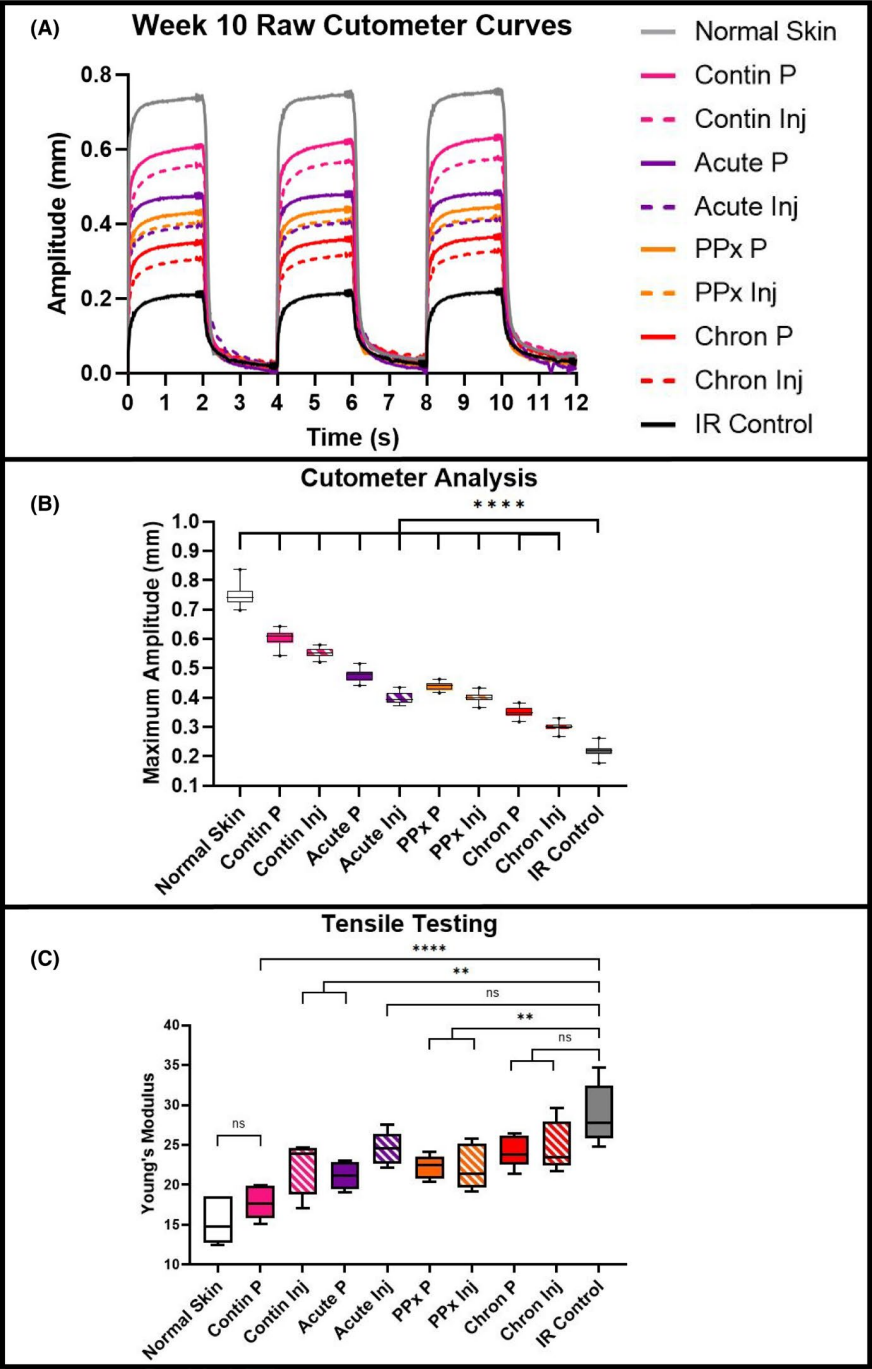
Biomechanical properties. (A) Representative suction Cutometer curves at week 10. One curve displayed for each group. (B) Elasticity analysis at week 10. Mean maximum suction amplitude was significantly higher for DFO treated groups compared with IR Control skin (****p < 0.0001), with greatest amplitude appreciated for continuous DFO patch treatment (Contin P). (C) Post‐harvest tensile testing revealed most DFO treatment groups were significantly less stiff than IR Control skin. Patch or injection treatment during post‐recovery chronic phase (Chron P and Chron Inj) and DFO injection during acute recovery (Acute Inj) groups had lower mean Young's Moduli vs. IR Control, however, none of these three groups achieved statistical significance like the other groups (**p < 0.01, ****p < 0.0001). Abbreviations: Chron, chronic; Contin, continuous; DFO, deferoxamine; Inj, injection; IR, irradiation; ns, not significant; P, patch; PPx, prophylactic

Paralleling histologic findings, weekly suction Cutometer measurements revealed significantly decreased dermal elasticity following radiation therapy compared with unirradiated skin. Treatment with DFO via patch delivery or injection showed a comparatively smaller decrease in elasticity than IR control skin (Figure 4A,B). However, while all DFO treatment regimens were associated with improved elasticity, the greatest benefit was appreciated with Contin P treatment. Finally, tensile testing of skin samples at week 10 matched Cutometer readings (Figure 4C). DFO treatment whether through patch or injection resulted in reduced stiffness when administered prophylactically, during acute recovery, or continuously. Nonetheless, continuous patch treatment yielded the greatest effect, with a Young's modulus similar to normal non‐irradiated skin (Figure 4C).

### 3.3 Skin perfusion

**FIGURE 5:**
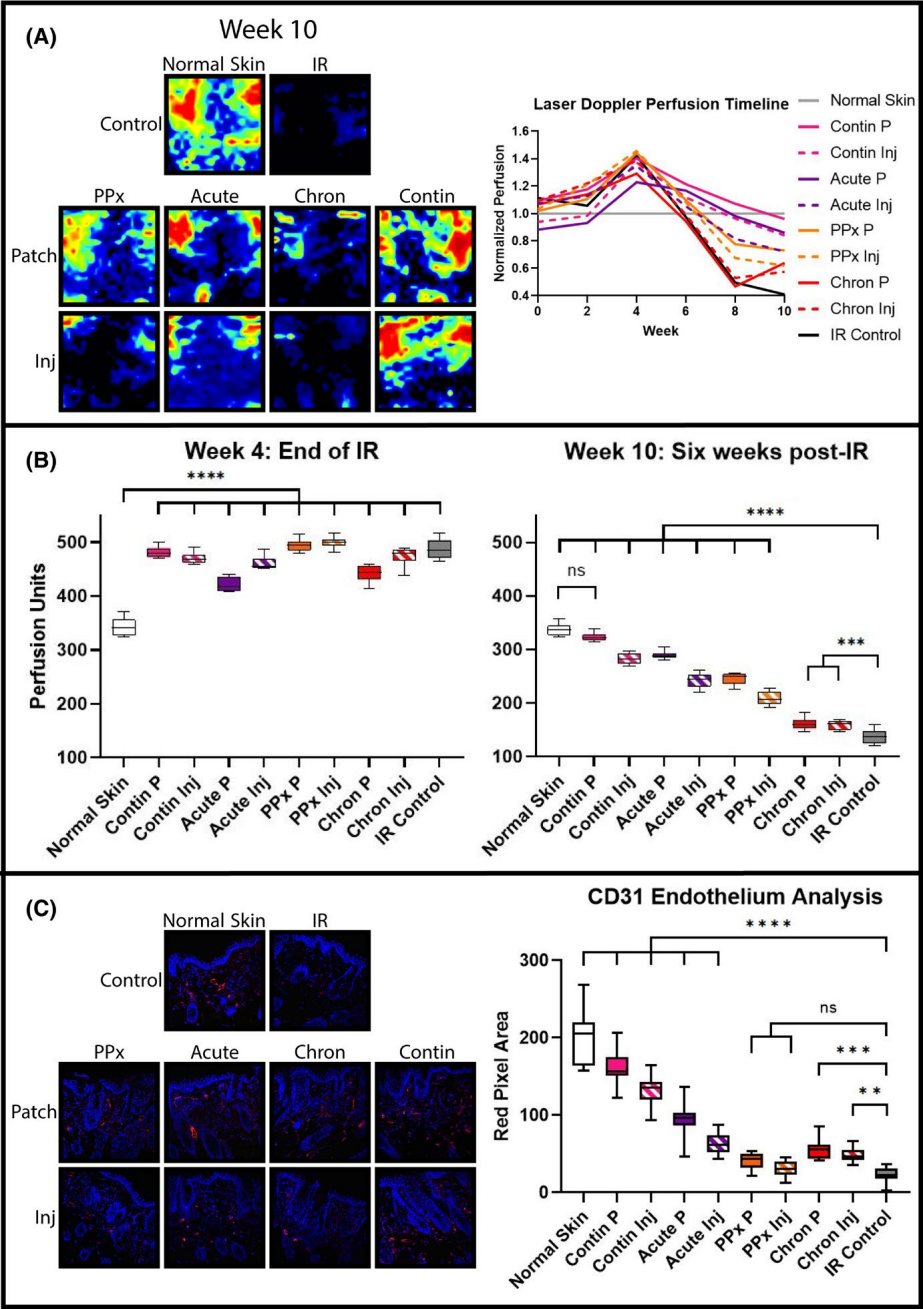
Dermal microvasculature analysis. (A) Laser Doppler perfusion imaging representative heat maps of dorsal skin at week 10 (left) and longitudinal perfusion tracking measured biweekly throughout experiment (right chart). (B) Mean perfusion analysis at week 4 (left chart) and week 10 (right chart). At week 4, immediate radiation injury elevated mean perfusion for all groups compared with Normal Skin (****p < 0.0001). By week 10, DFO treatment groups' mean perfusion levels decreased less than IR Control skin (***p < 0.001, ****p < 0.0001). The highest perfusion measured with continuous DFO patch treatment (Contin P) was noted, which was similar to normal skin. (C) Representative 20× CD31 immunofluorescent images (left) and quantification (right). Most DFO treatments increased immunofluorescent staining for CD31 compared with IR Control skin (**p < 0.01, ***p < 0.001, ****p < 0.0001). Note that, prophylactic DFO patch (PPx P) and prophylactic DFO injection (PPx Inj) groups' mean red pixel area was greater than IR Control; however, neither achieved significance. Abbreviations: Chron, chronic; Contin, continuous; DFO, deferoxamine; Inj, injection; IR, irradiation; ns, not significant; P, patch; PPx, prophylactic

Comparable to previous reports, longitudinal laser Doppler perfusion measurements revealed a significant decrease in perfusion in irradiated skin by week 8, the chronic injury phase, 4 weeks after completion of radiation. Initially, however, short‐term perfusion rose in response to IR, demonstrated by increased measurements compared with normal non‐radiated skin at week 4 (Figure 5A,B). Perfusion decreased in the following weeks for all irradiated groups, but DFO treated skin decreased less compared with IR control (Figure 5B). While all DFO treatment regimens were associated with increased laser Doppler perfusion readings at week 10 compared with IR control skin, Contin P treatment perfusion was most similar to normal skin.

CD31 immunofluorescent staining confirmed decreased vascularity in irradiated skin samples, and increased vascularity was appreciated in most DFO treated groups (Figure 5C). Delivery of DFO via patch or injection during acute recovery, in the post‐recovery chronic phase, or continuously, increased CD31 staining at week 10. However, the greatest vascularity was appreciated with continuous patch delivery, which again was the closest to normal skin histologically of all treatment groups.

## 4 DISCUSSION

### 4.1 Deferoxamine reduces indirect radiation damage by decreasing reactive oxygen species production

Iron chelation removes the catalyst for Fenton‐based ROS production. A decrease in indirect radiation injury is achieved as inflammatory cascades, secondary to the oxidative damage caused by these ROS, are inhibited from the outset. This can play a quite significant protective role, considering more tissue injury may occur via this indirect mechanism of radiation than by the initial direct DNA damage.^32^, ^33^ We investigated how DFO would affect this process by testing several downstream effects of pathologic oxidation. 8‐Iso, an arachidonic acid metabolite and isoform of naturally occurring prostaglandin F2alpha, is a product of lipid peroxidation. This is a more stable and reliable biomarker for oxidative damage compared with direct measurement of ROS themselves, which have fleeting half‐lives on the order of nanoseconds.^34^, ^35^, ^36^ In addition, the antioxidant enzyme, glutathione, exists in its oxidized by‐product form, GSSG, and in its reduced active form, GSH. The GSSG:GSH ratio is thus another indicator of oxidative stress level. Comparatively, more GSSG will be present in cases of greater ROS production since active GSH becomes oxidized while protecting cellular components.^37^ Finally, as ROS‐mediated damage accumulates, p53 tumour suppressor‐activated apoptotic proteins are expressed. One of these, BAX, is a pore‐forming protein responsible for mitochondrial cytochrome‐C release and functions as an apoptotic activator.^38^, ^39^, ^40^ In our study, iron chelation decreased lipid peroxidation, increased active antioxidant stores and decreased apoptotic cellular events in the dermis. Furthermore, while both patch delivery and injection of DFO were effective at reducing ROS, transdermal patch delivery showed a greater effect than direct injection for each of these oxidative damage measures.

### 4.2 Deferoxamine ameliorates dermal fibrosis secondary to radiation injury

Biomechanical changes indicative of fibrosis were also reduced with DFO treatment. In particular, normal‐appearing collagen content and extracellular matrix configurations were preserved with continuous patch DFO delivery. Longitudinal elasticity measurements and subsequent tensile testing were congruent with histological findings. DFO helped to maintain elasticity of skin, avoiding thickening and stiffening that would have occurred with expected RIF progression. For each biomechanical metric, the transdermal patch showed a greater effect than injection, and the greatest effects were appreciated with treatment during acute recovery or continuous therapy. Additionally, DFO treatment prophylactically or during the post‐recovery chronic phase still showed an effect, albeit to a lesser degree than the other two regimens. These findings may highlight the importance of timing for DFO‐mediated ROS abatement. Prophylactic DFO administration may have a lesser effect on subsequent radiation‐induced ROS generation, while post‐recovery chronic phase delivery may occur well beyond the key period of ROS generation and initiation of tissue damage.

### 4.3 Dermal blood supply is augmented by deferoxamine treatment

Laser Doppler imaging indicated that DFO treatment minimized the drop in perfusion usually seen with the development of fibrosis.^41^ Examination of these results along with CD31 immunofluorescent staining provided further insight into timing of delivery and administration route. Paralleling previously published work, injection of DFO in the post‐recovery chronic phase increased laser Doppler measured perfusion and histologic vascularity.^12^ Continuous patch treatment was also noted to yield the greatest effect, as similarly shown by Shen et al.^13^ Since endothelial damage is both a product of radiation injury and a catalyst of fibrosis development, preservation of perfusion may contribute, at least in part, to the improved fibrosis measures observed.^10^ With DFO treatment in the post‐recovery chronic phase, improvement in fibrosis may be more reliant on enhanced blood flow and the replenishment of a healthy microenvironment than on damage prevention. Of note, we observed an increase in perfusion early following radiation therapy. While this was not observed by Shen et al.,^13^ site‐specific differences in skin, as well as standardized conditions for measurement, may have contributed to this discrepancy. Furthermore, our findings in this present manuscript parallel histologic findings by others, showing acute radiation exposure increasing vascular permeability and transient pathologic blooming of irregular capillaries.^42^, ^43^

### 4.4 Transdermal delivery potentiates the anti‐fibrotic effects of deferoxamine treatment

This study revealed a greater benefit from transdermal DFO administration than from a solubilized injectable formulation. Although both were effective at reducing RIF, greater improvement was noted with the patch for each treatment regimen. Repetitive trauma alone, like in the case of these needlestick injuries, causes fibrosis. For example, lipodystrophy and similar fibroproliferative reactions are commonly seen in patients with diabetes who inject insulin without vigilant site rotation.^44^, ^45^, ^46^ Daily injections over the treatment duration (70 injections total in the case of the Contin Inj group) in the same area may cause significant injury and can be counterproductive to wound healing. In breaching the protective barrier of the skin, bacterial inoculation also becomes a possibility, and attendant inflammation could similarly promote fibrosis. Thus, even if the injectable form were to deliver a higher local concentration of DFO, it may come at a cost.

Additionally, the patch provided a more consistent release of DFO compared with injections. DFO's half‐life is quite short, on the order of hours, so an effective local concentration may not be maintained long after the cessation of treatment.^47^ The patches remained in place continuously, eluting DFO into the dermis in sustained fashion until the next day's patch was applied. In contrast, daily solubilized injectable DFO was given all at once. This made for an episodic dosing regimen, so although the same total amount of DFO was delivered, the dermal exposure to the drug may have been shorter lived. This may have, therefore, caused fluctuations in iron concentration between treatments which may have influenced downstream effects.

### 4.5 Future directions and limitations

There is great demand for an effective treatment for RIF. Treatments such as early rehabilitative care, laser therapy and hyperbaric oxygen therapy, as well as pharmacological treatments including pentoxifylline and superoxide dismutase may hold potential, however, studies examining the efficacy of these remain lackluster.^8^ Although our results in a murine model are promising, topical deferoxamine treatment needs further experimentation before it can be translated into clinical practice. Investigating the intracellular effects of DFO may provide further insight into the discrepancy in ROS measures we found between patch and injection groups. In addition to low penetrance of the stratum corneum, DFO cannot easily cross cellular/organelle lipid bilayers. Therefore, DFO introduced to the extracellular compartment should theoretically only chelate extracellular iron.^48^ The reverse micelle formulation may also be providing a means for DFO to enter the cell, where it can act upon intracellular iron. Mitochondria are responsible for the majority of intracellular ROS production, and reducing labile mitochondrial iron has been shown to alleviate cellular damage in ischaemia/reperfusion injury, another ROS‐mediated pathway of cytotoxicity.^19^, ^49^, ^50^ Moreover, several studies have implicated intracellular lysosomal iron in ROS‐mediated injury.^51^, ^52^

Importantly, mouse skin is different from human skin, and murine metabolism and healing/fibrosis occur more rapidly than with humans. Periods of recovery and the development of chronic fibrosis were, therefore, defined based on previously published studies.^53^, ^54^ Mouse skin layers also have different relative thicknesses compared with human skin. Additionally, a layer of subdermal muscle present in mouse skin, the panniculus carnosus, is absent in human skin.^55^ Therefore, similar studies in a large animal model such as pigs, where the skin is much more structurally similar to human skin, would be important for greater translational value of our findings.

## 5 CONCLUSION

Deferoxamine delivered directly to radiation‐damaged skin augments healing and decreases fibrosis. The transdermal patch formulation was more efficacious than the injectable form within each treatment regimen. A prophylactic effect was also observed, limiting self‐perpetuating ROS‐mediated inflammatory processes involved in fibrosis development.

## CONFLICT OF INTEREST

Dr. Michael Longaker and Dr. Geoffrey Gurtner have equity stakes in TauTona Group, the DFO patch supplier. Dr. Geoffrey Gurtner holds a patent for topical and transdermal HIF‐1 modulators for chronic wound treatment. Dr. Derrick Wan, Dr. Michael Longaker and Dr. Geoffrey Gurtner hold a patent for the use of DFO in conditioning irradiated tissue.

## AUTHOR CONTRIBUTIONS

**Christopher V Lavin:** Conceptualization (equal); Investigation (lead); Project administration (lead); Writing‐original draft (equal). **Darren B Abbas:** Investigation (equal); Writing‐original draft (equal). **Evan J Fahy:** Investigation (equal); Writing‐original draft (equal). **Daniel K Lee:** Formal analysis (equal); Investigation (equal). **Michelle Griffin:** Investigation (equal); Writing‐original draft (equal). **Nestor M Diaz Deleon:** Investigation (equal); Resources (equal). **Shamik Mascharak:** Formal analysis (equal). **Kellen Chen:** Formal analysis (equal); Writing‐original draft (equal). **Arash Momeni:** Supervision (equal); Writing‐review & editing (equal). **Geoffrey Gurtner:** Methodology (equal); Supervision (equal). **Michael T. Longaker:** Methodology (equal); Supervision (equal); Writing‐review & editing (equal). **Derrick Wan:** Conceptualization (equal); Supervision (lead); Writing‐review & editing (lead).

## Supporting information

Figure S1

## Data Availability

The data that support the findings of this study are available from the corresponding author upon reasonable request.
