# Supplementary figures and images for: A comparative analysis of deferoxamine treatment modalities for dermal radiation‐induced fibrosis
Source: J Cell Mol Med. 2021 Oct 6;25(21):10028–38. doi: 10.1111/jcmm.16913 (PMC8572785; doi:10.1111/jcmm.16913)

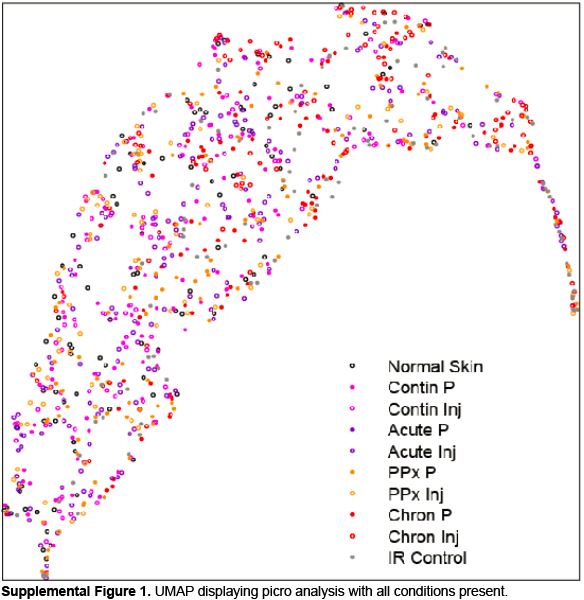

Supplement: Supplementary file 1 — Figure S1 [file JCMM-25-10028-s001.JPG]
